# Chemical defense of toad tadpoles under risk by four predator species

**DOI:** 10.1002/ece3.5202

**Published:** 2019-04-26

**Authors:** Bálint Üveges, Márk Szederkényi, Katharina Mahr, Ágnes M. Móricz, Dániel Krüzselyi, Veronika Bókony, Herbert Hoi, Attila Hettyey

**Affiliations:** ^1^ Lendület Evolutionary Ecology Research Group, Plant Protection Institute, Centre for Agricultural Research Hungarian Academy of Sciences Budapest Hungary; ^2^ Konrad Lorenz Institute of Ethology, Department of Integrative Biology and Evolution University of Veterinary Medicine Vienna Vienna Austria; ^3^ Department of Evolutionary Zoology and Human Biology University of Debrecen Debrecen Hungary; ^4^ Department of Pathophysiology, Plant Protection Institute, Centre for Agricultural Research Hungarian Academy of Sciences Budapest Hungary

**Keywords:** amphibia, bufadienolides, invertebrate predators, palatability, predator‐induced phenotypic plasticity, vertebrate predators

## Abstract

Many organisms use inducible defenses as protection against predators. In animals, inducible defenses may manifest as changes in behavior, morphology, physiology, or life history, and prey species can adjust their defensive responses based on the dangerousness of predators. Analogously, prey may also change the composition and quantity of defensive chemicals when they coexist with different predators, but such predator‐induced plasticity in chemical defenses remains elusive in vertebrates. In this study, we investigated whether tadpoles of the common toad (*Bufo bufo*) adjust their chemical defenses to predation risk in general and specifically to the presence of different predator species; furthermore, we assessed the adaptive value of the induced defense. We reared tadpoles in the presence or absence of one of four caged predator species in a mesocosm experiment, analyzed the composition and quantity of their bufadienolide toxins, and exposed them to free‐ranging predators. We found that toad tadpoles did not respond to predation risk by upregulating their bufadienolide synthesis. Fishes and newts consumed only a small percentage of toad tadpoles, suggesting that bufadienolides provided protection against vertebrate predators, irrespective of the rearing environment. Backswimmers consumed toad tadpoles regardless of treatment. Dragonfly larvae were the most voracious predators and consumed more predator‐naïve toad tadpoles than tadpoles raised in the presence of dragonfly cues. These results suggest that tadpoles in our experiment had high enough toxin levels for an effective defense against vertebrate predators even in the absence of predator cues. The lack of predator‐induced phenotypic plasticity in bufadienolide synthesis may be due to local adaptation for constantly high chemical defense against fishes in the study population and/or due to the high density of conspecifics.

## 1 INTRODUCTION

The ability of a genotype to produce different phenotypes in response to varying environmental conditions is known as phenotypic plasticity (Futuyma, 1998; West‐Eberhard, 1989, 2003). It became a central topic of evolutionary ecology because of its fundamental role in shaping biodiversity, ecological processes, and possibly even speciation (Agrawal, 2001; Miner, Sultan, Morgan, Padilla, & Relyea, 2005; Pfennig et al., 2010; West‐Eberhard, 1989, 2003). Inducible defenses are special cases of plastic responses, evoked by biotic environmental factors, such as predators (Harvell, 1990; Tollrian & Harvell, 1999), and can affect predator–prey interactions and hence prey survival probabilities. For example, animals are capable of changing their behavior, morphology, physiology, growth rate, and development speed as a response to predation risk (Harvell, 1990; Miner et al., 2005; Tollrian & Harvell, 1999; West‐Eberhard, 1989).

In natural environments, the density and composition of the predator fauna can vary immensely and unpredictably over time and space. Therefore, prey may considerably benefit from plastic adjustments in defensive traits. Because different types of predators can differ in their dangerousness, foraging strategy, microhabitat preferences, etc., different defensive responses may be effective against them. Accordingly, prey often respond to different predators with specific changes in behavior (Crowder, Squires, & Rice, 1997; Krupa & Sih, 1998; McIntosh & Peckarsky, 1999; Relyea, 2003; Turner, Fetterolf, & Bernot, 1999), morphology (Benard, 2006; Hoverman & Relyea, 2009; Kishida & Nishimura, 2005; Relyea, 2003) and life history (Relyea, 2003).

Chemical defenses have been mostly neglected in regard to phenotypic plasticity (Hettyey, Tóth, & Buskirk, 2014), even though they are widespread in the animal kingdom (Brodie, 2009; Mebs, 2001), and in many cases, toxin compounds have been identified and their effects on adversaries are well known (Blum, 1981; Mebs, 2001; Pawlik, 1993; Savitzky et al., 2012; Tachibana, 1988; Toledo & Jared, 1995). The few studies on inducible chemical defenses in animals showed that sessile invertebrates do respond to predation risk by increasing toxin levels (Ebel, Brenzinger, Kunze, Gross, & Proksch, 1997; Pohnert, 2004; Slattery, Starmer, & Paul, 2001; Thornton & Kerr, 2002), and some vertebrates respond similarly to environmental stressors such as human disturbance (Bucciarelli, Shaffer, Green, & Kats, 2017), contaminants (Bókony, Mikó, Móricz, Krüzselyi, & Hettyey, 2017), and conspecifics (Bókony, Üveges, Móricz, & Hettyey, 2018; Üveges et al., 2017). Whether predators induce toxin synthesis in vertebrate prey has remained controversial (Benard & Fordyce, 2003; Bucciarelli et al., 2017; Hagman, Hayes, Capon, & Shine, 2009; Üveges et al., 2017). It is plausible, however, that similarly to other defensive traits, prey individuals might adjust the composition or quantity of their defensive chemicals to the type of predators present, also because predator species may differ in their susceptibility to toxins (Gunzburger & Travis, 2005; Mohammadi et al., 2016; Ujvari et al., 2015). To the best of our knowledge, apart from an experiment on an octocoral (Thornton & Kerr, 2002), no study has addressed before whether and how predator‐induced plasticity in chemical defense of animals varies with the type of predators present in their environment.

Anuran amphibians are ideal model organisms for the study of phenotypic plasticity (Miner et al., 2005). Changes in physiology, behavior, morphology, and life‐history traits of many anuran species have been shown to be inducible by predatory cues (Kishida & Nishimura, 2005; Laurila, Pakkasmaa, Crochet, & Merilä, 2002; Van Buskirk, 2002b). Moreover, many anurans, including bufonid toads, rely on noxious skin secretions for protection against predators (Gunzburger & Travis, 2005; Savitzky et al., 2012; Toledo & Jared, 1995). Toxins of toads are likely responsible for the unpalatability of their eggs, hatchlings, and tadpoles to a wide variety of predators (Denton & Beebee, 1991; Henrikson, 1990; Kruse & Stone, 1984; Lawler & Hero, 1997; Peterson & Blaustein, 1991; Relyea, 2001a; Toledo & Jared, 1995). Poisoning by toads can cause severe symptoms, including nausea, vomiting, convulsions, hypertension, cardiac arrhythmia, or even death (Chen & Huang, 2013; Kamboj, Rathour, & Mandeep, 2013; Toledo & Jared, 1995). One of the main toxic compounds of toad skin secretion are cardiotoxic steroids called bufadienolides (Flier, Edwards, Daly, & Myers, 1980; Mebs et al., 2007; Toledo & Jared, 1995), which act by inhibiting Na^+^/K^+^ ATPases through attaching to the ouabain binding site of these enzymes (Flier et al., 1980; Lingrel, 2010; Pierre & Xie, 2006; Schoner & Scheiner‐Bobis, 2007). These compounds are synthesised by toads de novo (Chen & Osuch, 1969; Porto, Baralle, & Gros, 1972; Üveges et al., 2017) and are present in their tissues from a very early age on (Bókony et al., 2016, 2018; Mebs et al., 2007; Ujszegi, Móricz, Krüzselyi, & Hettyey, 2017; Üveges et al., 2017).

**Figure 1:**
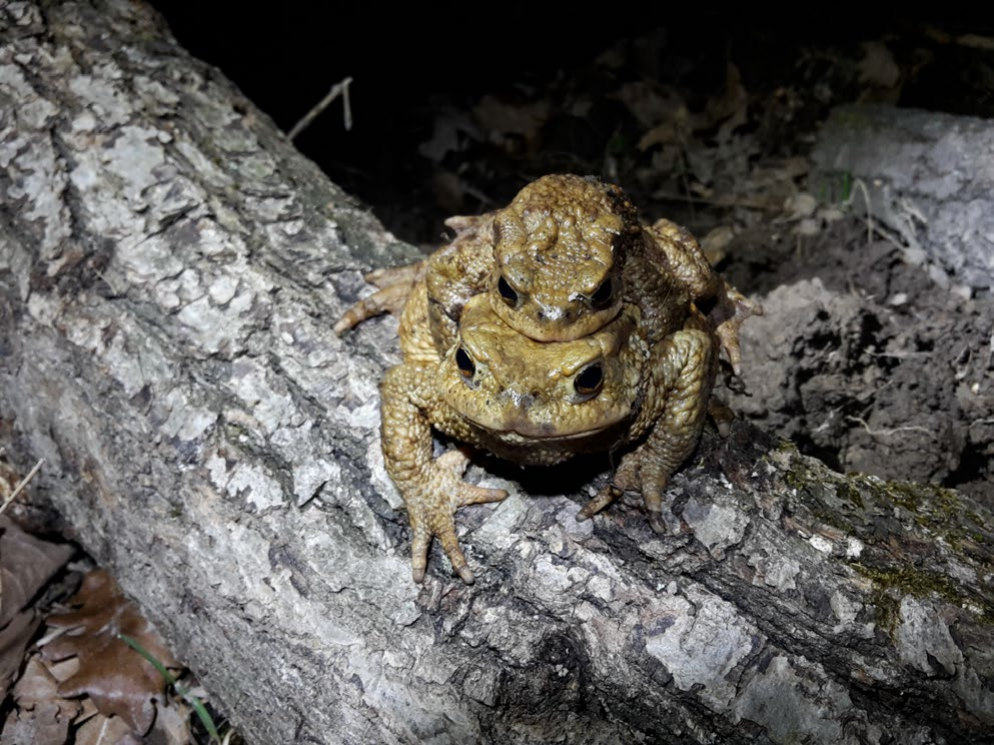
Amplexing pair of adult common toads (*Bufo bufo*). ©Bálint Üveges

Only a handful of studies tested so far if the bufadienolide synthesis of toads is inducible by predation risk (Benard & Fordyce, 2003; Hagman et al., 2009; Marion, Fordyce, & Fitzpatrick, 2015; Üveges et al., 2017). However, these former experiments provided inconclusive results. Adult American toads (*Anaxyrus*, formerly *Bufo*,* americanus*) did not respond to repeated manual expression of their parotoid glands with changes in their chemical defense (Marion et al., 2015). Furthermore, bufadienolide content of common toad (*Bufo bufo*; Figure 1) tadpoles was not affected by predator cues (Üveges et al., 2017), even though plasticity in toxin production has been documented in the same study system in response to several other environmental stressors (Bókony et al., 2017, 2018; Üveges et al., 2017). Two studies found plastic responses to larval predation risk after metamorphosis, either as a change in the amount of bufadienolides in western toads (*Anaxyrus*, formerly *Bufo*, *boreas*; Benard & Fordyce, 2003) or in the size of the toxin‐producing parotoid glands in cane toads (*Rhinella marina*, formerly *Bufo marinus*; Hagman et al., 2009). However, neither study demonstrated predator‐induced changes in chemical defense during the larval stage when tadpoles were exposed to predator cues. Furthermore, only one study (Benard & Fordyce, 2003) investigated whether inducible toxin production can enhance survival probability of toads when exposed to predators, but the effects observed in postmetamorphic toads were counterintuitive. One potential reason for the controversy of these previous findings may be that each experiment applied a single type of predatory cue, and some predators might induce stronger responses than others (Hettyey, Vincze, Zsarnóczai, Hoi, & Laurila, 2011; Hettyey, Zsarnóczai, Vincze, Hoi, & Laurila, 2010; Relyea, 2001b, 2003).

In this study, we investigated whether tadpoles adjust their chemical defenses to predation risk in general and specifically to the presence of four, phylogenetically distant predator species. In addition, we also assessed the adaptive value of the induced defense. To accomplish these goals, we reared common toad tadpoles in outdoor mesocosms in the presence or absence of caged predators, measured their bufadienolide content, and finally assessed their survival upon exposure to free‐ranging predators. We chose the common toad as our study species, because its tadpoles display relatively weak plastic responses to predators during the larval stage in terms of morphology and behavior (Lardner, 2000; Laurila, Kujasalo, & Ranta, 1998; Van Buskirk, 2002a), but appear to be unpalatable to several predator species (Denton & Beebee, 1991; Henrikson, 1990), suggesting a heavy reliance on chemical defense. We predicted that tadpoles raised with caged predators would contain an elevated number of bufadienolide compounds and/or larger total bufadienolide quantity than their predator‐naïve conspecifics. Also, we expected the strength of these responses to vary according to the predator species present. Tadpoles can assess the dangerousness of predators based on olfactory cues (Brönmark & Hansson, 2000; Hettyey et al., 2015; Kats & Dill, 1998). Depending on such cues, they should upregulate their toxin synthesis as a response to predators against which an increased allocation into chemical defense enhances unpalatability. Therefore, we expected the strongest response to predators that are voracious consumers of amphibian larvae in general, but are susceptible to bufadienolides, that is, vertebrates and especially fish (Gunzburger & Travis, 2005). On the other hand, we expected weak responses to predators that consume fewer tadpoles in total but can bypass the main reservoir of bufadienolides, the skin (Halliday et al., 2009; Toledo & Jared, 1995), by using a pierce and suck feeding mechanism (e.g., Heteroptera such as backswimmers). Finally, we predicted that tadpoles exhibiting predator‐induced phenotypes would have elevated survival probabilities compared to predator‐naïve conspecifics when facing free‐ranging predators.

## 2 MATERIALS AND METHODS

### 2.1 Ethics statement

Permits for collection and transport of animals were issued by the City of Vienna (MA22–120657/2014) and by the Land Niederösterreich (RU5‐BE‐7/016‐2014). Experimental procedures were approved by the institutional ethics committee and the national authority according to § 8ff of Law for Animal Experiments, Tierversuchsgesetz—TVG (GZ 68.205/0164‐II/3b/2013).

### 2.2 Experimental procedures

**Figure 2:**
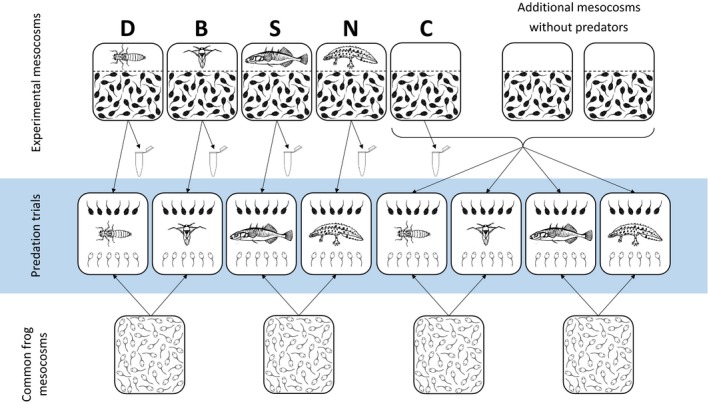
Schematic diagram of the experimental design, showing the experimental units on the example of a hypothetical common toad family. Upper, middle, and lower units represent mesocosms of focal toad tadpoles, predation‐trial tubs, and mesocosms of naïve frog tadpoles, respectively. Abbreviations represent predator treatments as follows: B: backswimmer, C: control; D: dragonfly larva; N: newt, S: stickleback. Each microcentrifuge tube represents two toads sampled for toxin analysis (one during the tadpole stage and one at the start of metamorphosis). Animal drawings by Viktória Verebélyi

We performed the experiment at the school area of PNMS/PHS Sacré Coeur in Pressbaum, Austria (48°11′11.32″N, 16°4′48.05″E), during spring 2014. We set up mesocosms ca. two weeks prior to the addition of toad eggs by filling plastic containers (82 × 58 × 30 cm, length × width × height) with 130 L tap water and adding 50 g dried beech (*Fagus sylvatica*) leaves to provide shelter for tadpoles and substrate for algal and microbial growth. Two days later, we inoculated each mesocosm with 1 L pond water containing phytoplankton and zooplankton. To prevent colonization by predators, we covered the containers with mosquito nets. Mesocosms were arranged in a full‐factorial randomized block design in which each block corresponded to one family of toads (see below). In each block, each experimental treatment was represented once (totaling 12 replicates for each treatment; Figure 2). Each mesocosm contained a cage in which we introduced a predator (except in the control treatment) one day before placing toad eggs into the mesocosms, as detailed below. Two further mesocosms per block containing an empty cage (i.e., no predator) served for raising additional predator‐naïve tadpoles for the predation trials (as detailed below; Figure 2).

We captured 12 amplexing pairs of common toads at Silbersee, Vienna, Austria (48°12′32.72″N, 16°15′47.61″E) and transported them to the site of the experiment. Furthermore, we also collected freshly laid common frog (*Rana temporaria*) eggs from a small pond near the site of the experiment (48°11′1.92″N, 16°4′40.87″E). We allowed toad pairs to lay eggs in 45‐L plastic containers placed outdoors, containing twigs as egg deposition substrates, and filled with ca. 15 L aged tap water. On the day when the last pair finished egg deposition, we randomly assigned ca. 120 developing eggs from each clutch to a given mesocosm and placed them into a plastic dish equipped with a mesh bottom floating on the water surface. This way, developing embryos were already in contact with chemical cues present in the mesocosms. Captive pairs laid their eggs within 6 days, but developmental differences among clutches were not detectable upon hatching (pers. obs.). Three weeks after egg laying, when tadpoles reached the free‐swimming state (developmental stage 20 according to Gosner, 1960), we haphazardly selected 60 healthy‐looking individuals from each plastic dish and released them into the open water of the corresponding mesocosm (day 1 of the experiment). We removed remaining tadpoles and the plastic dishes from the mesocosms.

To simulate predation risk by predators that may find toad tadpoles diversely palatable, we collected late‐instar larvae of the southern hawker (*Aeshna cyanea*, hereafter dragonfly), adult backswimmers (*Notonecta* sp.), and adult male smooth newts (*Lissotriton vulgaris*) from private ponds in Austria, and acquired juvenile three‐spined sticklebacks (*Gasterosteus aculeatus*) from a breeder. We obtained all predators before the start of the breeding season of common toads. Predators were housed in horizontally oriented, partially submerged cages, one cage per mesocosm, made from PVC tubes (21 × 11 cm, length × diameter), both ends covered with a double layer of mosquito netting. We fed each predator three times a week with one common toad and one common frog tadpole. The use of both toad and common frog tadpoles as prey items closely models a natural scenario, because these species often co‐occur in ponds in the study area (pers. obs.). Moreover, tadpoles are able to differentiate between predation cues from feeding on conspecifics or heterospecifics (Hettyey et al., 2015). Therefore, by feeding predators both prey species, the focal tadpoles could have received information about how dangerous the predator is to tadpoles in general, and also on its willingness to feed on toad tadpoles. Both kinds of information are important in determining the necessity to upregulate the synthesis of bufadienolides. One would expect that predators which are readily deterred by baseline levels of defensive chemicals or against which toxins are ineffective should not induce an increase in bufadienolide levels. In the former case, an upregulation of toxin synthesis would be unnecessary, while in the latter case it would be useless, or achieving a toxin level that can overcome the predator's resistance may be physiologically and/or energetically unfeasible.

We kept prey tadpoles of both species in mixed‐family groups in separate containers that provided similar conditions for them as for focal tadpoles, but without the presence of any predator cues. On each feeding occasion, we removed cages from the mesocosms, documented the number of surviving and consumed tadpoles since the last feeding event, replaced them with new ones, and put the cages back into the water. To ensure uniform disturbance, we handled control (empty) cages in the same way, but without introducing tadpoles. When a predator died or did not eat for two consecutive feeding occasions, we replaced it with a new conspecific (substitute predators were kept in the same manner as specimens for the predation trials, see below). Survival of three predator species was high during the whole study: 20 out of 20 (100%) dragonfly larvae, 14 out of 17 (82.35%) sticklebacks, and 39 out of 40 (97.5%) newts survived. However, out of 64 backswimmers only 14 (21.88%) survived (varying total numbers arise from replacements of fasting or dead individuals).

To assess chemical defenses of toad tadpoles, we collected samples on two occasions by preserving tadpoles in 70% HPLC grade methanol (Figure 2). First, we haphazardly selected one individual from each mesocosm thirteen or fourteen days after start of the experiment (developmental stage 29, *N* = 60; sampling lasted for two days because we also photographed tadpoles and measured their body mass, see Appendix S1). Second, we preserved the 10th toad tadpole to start metamorphosis (developmental stage 42) from each mesocosm (*N* = 60). Additional mesocosms that served to raise predator‐naïve tadpoles for the predation trials (Figure 2) were not sampled on either occasion.

During the experiment, we collected further data on tadpole behavior, body mass, morphology, length of larval development, and survival (for detailed methodology and results see Appendix S1). We investigated these variables to analyze whether predator cues induced any phenotypic change other than chemical defense, because such changes might have influenced the outcome of the predation trials. Adult toads, remaining tadpoles, and predators, apart from sticklebacks, were released at their site of origin as soon as possible. Remaining sticklebacks were released into a private garden pond in Pressbaum, Austria.

### 2.3 Predation trials

The aim of this experiment was to test whether the presence of predatory cues during the rearing stage increased survival of common toad tadpoles when facing free‐swimming predators. Therefore, we housed additional 24 specimens of each predator species separately during the study. We kept dragonfly larvae and backswimmers individually in 1 L (container size: 18 × 13 × 12 cm) and 3 L (29 × 19 × 14 cm) aged tap water, respectively, whereas sticklebacks and newts in groups of 12 in 40 and 20 L aged tap water, respectively (57 × 39 × 28 cm). Housing tubs of dragonfly larvae and backswimmers were equipped with a perching stick. We fed these predators three times a week ad libitum with *Tubifex* sp. (all predators), bloodworms (*Chironomus* sp.; dragonfly larvae), white mosquito larvae (*Chaoborus* sp.; backswimmers), and white worms (*Enchytraeus* sp.; sticklebacks and newts). To make predators accustomed to eating tadpoles, four and two days before the start of the predation trials (day ten and twelve) each predator received a toad and a frog tadpole. Predators were provided with toad tadpoles at these two feeding occasions from the respective rearing container to which the given individual was a priori randomly assigned to.

To set up predation‐trial venues, on day two of the main experiment we filled 45‐L plastic tubs with 40 L aged tap water and added 0.3 L pond water and 9 g dried beech leaves to provide food and shelter for tadpoles. Eleven days later (day thirteen), we placed six toad tadpoles into each predation‐trial tub, accompanied by six predator‐naïve common frog tadpoles as alternative prey (see below, Figure 2). Toad tadpoles were haphazardly chosen from the experimental rearing tubs and were assigned to a predation‐trial tub that would contain the same predator species they had been raised with (Figure 2). For each predator species and each toad family, we used two predation‐trial tubs: we introduced six toad tadpoles that had been raised with predators into one of the tubs, and we placed six predator‐naïve control toad tadpoles into the other tub (Figure 2). This resulted in 96 predation‐trial tubs (4 predator species × 2 toad tadpole treatments, i.e., raised with or without a predator × 12 families). We used this approach to easily distinguish between tadpoles raised with and without predators, as other identification techniques (e.g., implant tags) were not logistically feasible at the time. After a 24‐hr acclimatization period for tadpoles (on day fourteen), we released the assigned predator into each predation‐trial tub. Note that the predator individuals used in these trials were not the same as the individuals used in the rearing tubs. Predators were fasted for 2 days before the trial. Given that the four species of predators differ in voraciousness (Table 1), we determined the duration of the trials separately for each species (dragonfly larvae: 30 hr, backswimmers: 48 hr, sticklebacks: 84 hr, newts: 120 hr) by monitoring the predation‐trial tubs and terminating all trials involving a given type of predator when approximately half of all the tadpoles were eaten. After termination, we counted survivors of both tadpole species and assessed body size of predators by measuring wing length (dragonfly larvae), body length (backswimmers and sticklebacks), or snout–vent length (newts) to the nearest 0.01 mm using a digital calliper.

**Table 1:** Percentage of tadpoles consumed by predators over the feeding sessions in the cages suspended in the mesocosms during the rearing period of the experiment

Predator species	% Toad larvae	% Frog larvae	Estimate	*SE*	*t*	*p*
Dragonfly larvae	91.18 ± 2.41	93.71 ± 2.39	0.364	0.349	1.043	0.308
	(80.77–100)	(84.62–100)				
Backswimmer	73.66 ± 3.05	89.94 ± 3.18	1.166	0.253	4.607	<0.001
	(62.96–88)	(74.07–100)				
Stickleback	32.51 ± 6.33	94.5 ± 2.83	3.602	0.457	7.889	<0.001
	(10.34–60.71)	(80.77–100)				
Smooth newt	6.53 ± 1.75	72.77 ± 4.4	3.656	0.283	12.94	<0.001
	(0–15.38)	(57.69–89.29)				

Note

Mean ± *SE* and range (in brackets), as well as the results of generalized linear model with quasibinomial distribution comparing the survival of toad and frog tadpoles, are presented. Estimates represent the difference in logit survival between toad and frog tadpoles.

We introduced frog tadpoles into the predation‐trial tubs because our aim was to test the utility of chemical defense in an ecologically relevant scenario where predators can choose among different prey. Also, predators are less discriminative and more likely to prey on chemically defended organisms when hungry than when satiated (Barnett, Bateson, & Rowe, 2007; Gillette, Huang, Hatcher, & Moroz, 2000; Hileman, Brodie, & Formanowicz, 1994; Kruse & Stone, 1984; Sandre, Stevens, & Mappes, 2010). Therefore, without the presence of alternative food source, that is, frog tadpoles, predators may have had consumed highly defended toads (reared with predatory cues) and poorly defended ones (controls) at similar rates, and thus, the effect of hunger would have confounded our results (Gunzburger & Travis, 2005). Finally, this design also enabled us to measure differences in voraciousness between individual predators and to control for these differences in the statistical analyses (by including the number of frog tadpoles eaten as a covariate, see below). This was necessary because each predator received toad tadpoles that were either raised with or without predatory cues (i.e., survival differences between naïve and treated tadpoles might arise if systematically more voracious individuals are accidentally assigned to one treatment group). Toad and frog tadpoles introduced into the predation trials were of somewhat different size (mean body mass ± *SE*; toads: 163.81 ± 2.04 mg, frogs: 121.81 ± 3.28 mg, based on subsamples of 58 individuals per species). Nonetheless, these size ranges correspond to relatively young tadpoles of small to intermediate size in these species; therefore, it is unlikely that they posed a problem even for gape‐limited predators, such as sticklebacks and newts (Eklöv & Werner, 2000; Richards & Bull, 1990; Semlitsch & Gibbons, 1988; Wilson & Franklin, 2000).

### 2.4 Chemical and statistical analyses

Preparation of samples and analysis of bufadienolide content of toads was carried out using high‐performance liquid chromatography with diode‐array detection and mass spectrometry (HPLC‐DAD‐MS) according to an already published protocol (Üveges et al., 2017). Toxin content of tadpoles was assessed using three variables: number of bufadienolide compounds (NBC), total bufadienolide quantity (TBQ), and mass‐corrected total bufadienolide quantity (mcTBQ). When determining NBC for each animal, we considered a compound to be present when its signal to noise ratio was at least 3 in the HPLC‐MS chromatogram. We estimated the quantity of each compound from the area values of chromatogram peaks based on the calibration curve of the bufotalin standard and summed up these values to obtain estimates of TBQ for each individual. This approach yields approximate estimates of bufadienolide quantities and has been used in similar studies (Benard & Fordyce, 2003; Bókony et al., 2016, 2018; Hagman et al., 2009; Üveges et al., 2017). We calculated mcTBQ by dividing TBQ by the dry mass of individuals. TBQ measures the total toxin content of tadpoles, relevant for anti‐predatory defense, whereas mcTBQ represents the relative amount of resources allocated into toxin synthesis.

We analyzed the effects of predator treatment on toxin content using linear mixed‐effects models (LMM). We entered NBC, TBQ, or mcTBQ separately as the dependent variable. In case of NBC and TBQ, initial models included treatment and age of tadpoles (developmental stage 29 or 42) as fixed factors, dry mass as a covariate, and all two‐way interactions and the three‐way interaction. In case of mcTBQ, the initial model included only treatment and age as fixed factors and their two‐way interaction. In all models, mesocosm nested within family were included as random factors. We applied stepwise backward model simplification based on *p*‐values with *α* = 0.05 (Grafen & Hails, 2002). However, since the results of full and simplified models were qualitatively identical, we present statistics obtained from full models. We ran all analyses in R 3.4.0 (R Development Core Team, 2017) using the “lme” function in the “nlme” package (Pinheiro, Bates, DebRoy, Sarkar, & R Core Team, 2017). *p‐*values were calculated with the “ANOVA” function in the “car” package (Fox & Weisberg, 2011), using type‐2 sums of squares as suggested by Langsrud (2003) and Hector, Felten, and Schmid (2010) for models with interactions. Two samples were discarded from these analyses, because their dry mass was measured incorrectly (see Appendix S2). Additionally, we described the within‐individual diversity of bufadienolide compounds by applying hierarchical diversity partitioning using the “hierDiversity” package (Marion et al., 2015); for further information on this approach, see Appendix S1.

We analyzed survival of toad tadpoles in the predation trials for each predator species separately using generalized estimation equations (GEE) models. We chose this approach because the effect of predator size could not be modeled adequately using LMM with these data, that is, we wanted to control for the effect of predator size across all individuals (a population averaged effect, as estimated in GEE) and not within random factor levels (Zuur, Ieno, Walker, Saveliev, & Smith, 2009). As the dependent variable, we entered the proportion of toad tadpoles surviving out of all toad tadpoles in the predation‐trial tub. Initial models included toad tadpole treatment (i.e., predator‐naïve or raised with caged predator) as a fixed factor and the number of frog tadpoles eaten during the predation trial and predator size (to control for potentially different voraciousness between predators) as covariates. All models included toad family as the random factor. We ran analyses using the “geeglm” function in the R package “geepack” (Venables & Ripley, 2002) with binomial error distribution. We performed model simplification as described in the case of toxin content, but since there were no factors with more than two categories in these models, we evaluated the *p*‐values using the “summary” function in “geepack.” Confidence intervals for the survival estimates in the two treatment groups were calculated from the final models using the “lsmeans” function in the “lsmeans” package (Lenth, 2016). Only one newt ate a toad tadpole; therefore, we did not perform a formal analysis of survival in the presence of free‐ranging newts. Further, two backswimmers, one dragonfly, and one stickleback did not consume any tadpoles (neither toads nor frogs, see Appendix S2). Consequently, we could analyze survival in the remaining 22 trials involving backswimmers, 23 trials involving dragonfly larvae, and 23 trials involving sticklebacks.

Additionally, we also compared the mortality of naïve toad and naïve frog tadpoles fed to the caged predators during the rearing stage of the experiment using generalized linear models with quasibinomial distribution (Table 1). We ran a model for each predator separately. We included the proportion of tadpoles eaten out of all presented tadpoles as the dependent variable and species of tadpoles (toad or frog) as a fixed factor. We ran the analyses using the “glm” function in the “stats” package (R Development Core Team, 2017), and we used the “summary” function to calculate *p*‐values.

## 3 RESULTS

### 3.1 Antipredator responses of toad tadpoles

During the rearing stage of the experiment, dragonfly larvae emerged as the most voracious predator of toad tadpoles, followed by backswimmers, sticklebacks, and newts, in this order (Table 1). Furthermore, dragonfly larvae consumed both toad and frog tadpoles at similar rates, whereas all other predators preferred frogs over toads (Table 1). Nevertheless, all four species were voracious predators of frog larvae (Table 1).

**Figure 3:**
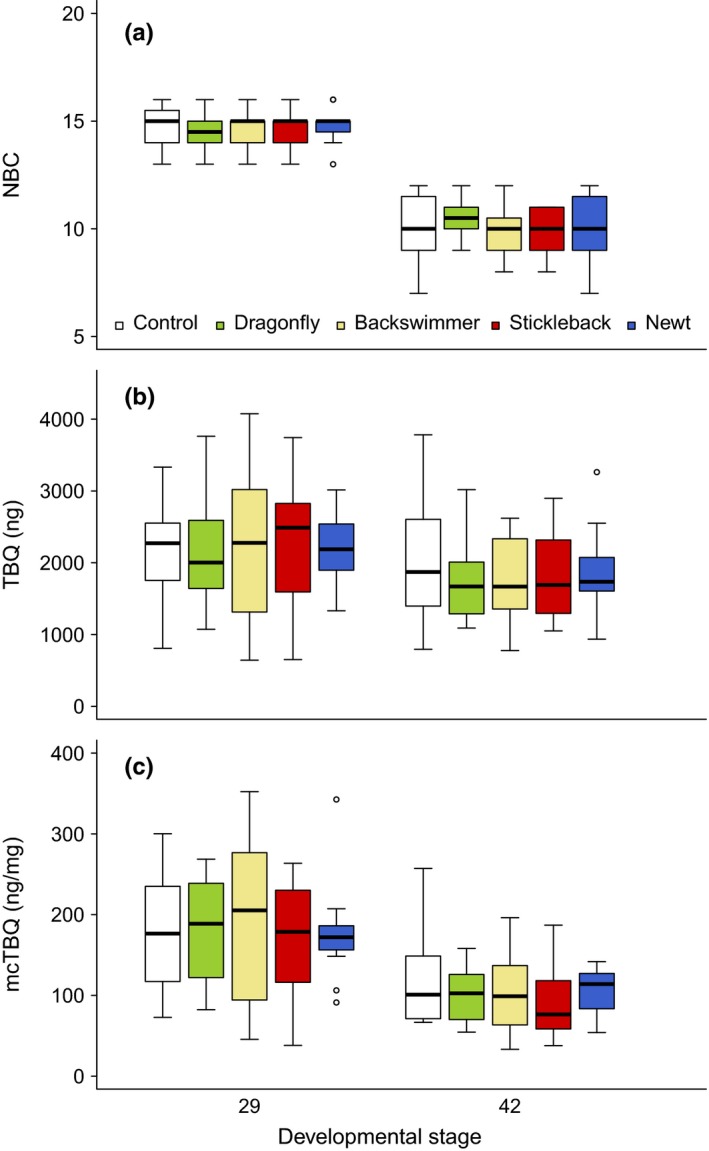
Toxin content of toads in the five predator‐treatment groups ca. midway through larval development (developmental stage 29) and at the onset of metamorphosis (developmental stage 42). (a) Number of bufadienolide compounds. (b) Total bufadienolide quantity. (c) Mass‐corrected total bufadienolide quantity. Thick horizontal lines and boxes represent the medians and interquartile ranges, respectively; whiskers extend to the upper and lower quartile ± 1.5 × interquartile range; open circles represent extreme data points

Predator treatments had no significant effect on total bufadienolide quantity (Table 2, Figure 3) or on the within‐individual diversity of bufadienolides (Figure S1). However, the interaction of tadpole dry mass and predator treatment had a significant effect on the number of bufadienolide compounds (Table 2): heavier tadpoles raised in the presence of sticklebacks had fewer bufadienolide compounds than expected from the allometric relationship between dry mass and NBC of control tadpoles (Table S1, Figure S2). The other three predator species had no significant effect on NBC (Table S1, Figure 3). Furthermore, compared to individuals that started metamorphosis (developmental stage 42), young tadpoles (developmental stage 29) had on average a 39.36% (mean ± *SE* of difference: 5.02 ± 0.23) higher NBC, 15.54% (340.83 ± 115.07 ng) higher TBQ and 42.26% (75.75 ± 9.32 ng/mg) higher mcTBQ (Table 2, Figure 3).

**Table 2:** Effects of age, dry mass, predator treatment, and their interactions on bufadienolide toxin content of common toad tadpoles

	*χ* ^2^	*df*	*p*
Number of bufadienolide compounds (NBC)			
Age	**481.847**	**1**	**<0.001**
Dry mass	**7.643**	**1**	**0.006**
Treatment	2.055	4	0.726
Age × dry mass	2.014	1	0.156
Age × treatment	3.276	4	0.513
Dry mass × treatment	**13.095**	**4**	**0.011**
Age × dry mass × treatment	3.744	4	0.442
Total bufadienolide quantity (TBQ)			
Age	**10.341**	**1**	**0.001**
Dry mass	2.139	1	0.144
Treatment	1.013	4	0.908
Age × dry mass	1.233	1	0.267
Age × treatment	0.778	4	0.941
Dry mass × treatment	1.073	4	0.899
Age × dry mass × treatment	1.571	4	0.814
Mass‐corrected total bufadienolide quantity (mcTBQ)			
Age	**62.605**	**1**	**<0.001**
Treatment	1.743	4	0.783
Age × treatment	1.206	4	0.877

Note

We present analysis of deviance tables with type‐2 sums of squares for the full linear mixed‐effects models. Significant terms are highlighted in bold.

We found no significant effect of predator treatment on behavior, body mass, or morphology of toad tadpoles and on their survival in the rearing mesocosms (Appendix S1). Time to metamorphosis was significantly shorter in the presence of sticklebacks than in control tubs (Figure S3), whereas the other three predator species did not affect the length of larval development (Appendix S1).

### 3.2 Predation trials

**Figure 4:**
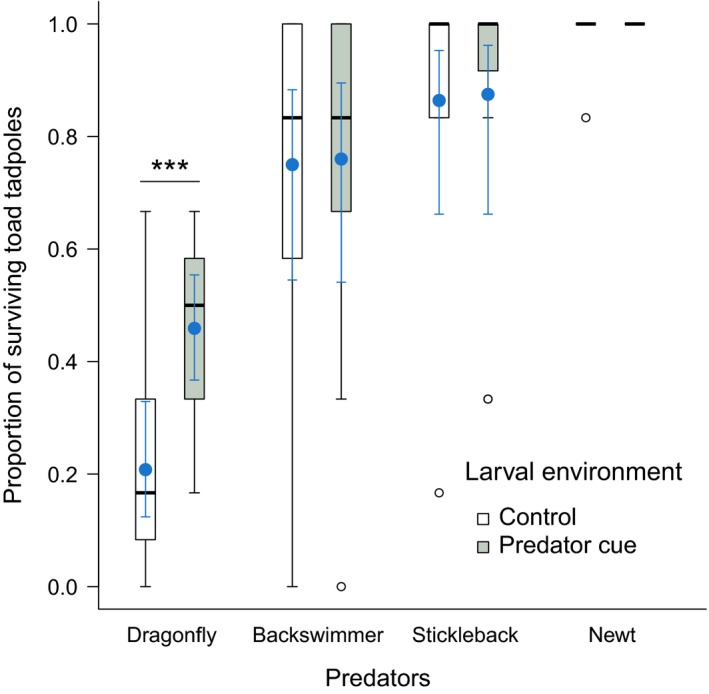
Proportion of surviving toad tadpoles in the predation trials, in relation to the treatment experienced during larval development. A significant difference is marked with asterisks (*p* < 0.001). For the interpretation of box plots, see Figure 3. Filled circles and error bars represent means ± 95% confidence intervals calculated from GEE models

When exposed to free‐ranging dragonfly larvae, toad tadpoles that developed in the presence of caged specimens of this predator had on average 25.1% higher survival compared to their predator‐naïve conspecifics (Table 3, Figure 4). The presence of caged backswimmers, sticklebacks, and newts during tadpole development did not have a significant effect on toad tadpole survival in predation trials (Table 3, Figure 4).

**Table 3:** Effects of treatment, predator size, and the number of common frog tadpoles eaten on survival of toad tadpoles in the predation trials

	*N*	Estimate	*SE*	Wald *χ* ^2^	*p*
Dragonflies					
Intercept	23	−0.501	0.642	0.610	0.440
Frog tadpoles eaten		−0.069	0.141	0.240	0.620
Predator size		−0.083	0.067	1.530	0.220
Treatment		**1.125**	**0.256**	**19.330**	**<0.001**
Backswimmers					
Intercept	22	−5.300	3.829	1.916	0.166
Frog tadpoles eaten		0.363	0.397	0.835	0.361
Predator size^*^		0.335	0.191	3.065	0.080
Treatment		0.725	0.629	1.331	0.249
Sticklebacks					
Intercept	23	6.505	5.036	1.670	0.200
Frog tadpoles eaten		0.195	0.267	0.540	0.460
Predator size		−0.119	0.115	1.070	0.300
Treatment		0.300	0.796	0.140	0.710

Note

We present the parameter estimates (±*SE*) of the full GEE models; the “intercept” shows the logit of survival for the control tadpoles, and the “treatment” parameter shows the difference in logit survival between the tadpoles raised with the respective predator and the control tadpoles. A significant effect is highlighted in bold, and a marginally nonsignificant effect is marked with an asterisk. We did not analyze predation trials involving newts because overall only one of these animals consumed a toad tadpole.

## 4 DISCUSSION

We found no evidence that common toad tadpoles respond to the presence of four different predator species by upregulating their bufadienolide synthesis. This finding agrees with results of earlier studies, which found no plastic changes in bufadienolide content of tadpoles of various toad species in response to predator cues (Benard & Fordyce, 2003; Üveges et al., 2017). However, when toad tadpoles were raised with predator cues, differences in chemical defenses between control and predator‐exposed individuals became apparent after metamorphosis (Benard & Fordyce, 2003; Hagman et al., 2009). This suggests that toads do respond to larval predation risk by some physiological changes in the bufadienolide synthesis pathway or anatomical changes in toxin‐producing structures that become detectable only during or after metamorphosis. Putting these findings together, it may be possible that tadpoles are unable to fine‐tune their toxin content, with the necessary regulatory mechanisms developing only at or after metamorphosis. However, previous results reject this explanation by demonstrating plastic adjustment of larval bufadienolide production in response to a variety of environmental factors, such as restricted food levels (Üveges et al., 2017), a herbicide (Bókony et al., 2017), and competitors (Bókony et al., 2018). Thus, it seems that toad tadpoles are physiologically capable of responding to stressors by upregulating their toxin levels, but we did not observe this pattern in response to predation risk.

A possible explanation for the lack of predator‐induced plasticity is that such plasticity may not always be adaptive. For example, when predation risk is permanent, any individual that fails to defend itself has little chance to survive. In such an environment, a plastic response may evolve into a constitutive defensive strategy, which is constantly expressed no matter the actual predator assemblage. Such fixation of an originally plastic trait is possible through genetic assimilation (Crispo, 2007; Pfennig et al., 2010; West‐Eberhard, 2003) in environments where a relevant inducing biotic or abiotic factor is persistent. This is a likely explanation in our case, given that the toad tadpoles used in the current study originated from a permanent pond inhabited by fishes. Because fishes have persisted for generations in this aquatic habitat, and they are one of the most voracious predators of amphibian larvae (Wells, 2007), it is possible that selection acted to reduce plasticity in bufadienolide synthesis in this population and favoured instead the maintenance of high toxin levels irrespective of the actual cues on predation risk. The same idea might explain the lack of predator‐induced plasticity in bufadienolide content in our previous experiment (Üveges et al., 2017).

Another environmental factor which may explain the lack of plastic antipredator responses in toxin production is the presence of conspecifics. A recent study showed that increased conspecific density can induce elevated bufadienolide synthesis in toad tadpoles (Bókony et al., 2018). Because in the present study tadpoles were reared at relatively high densities (1 tadpole/2.2 L water at the beginning of the experiment and 1 tadpole/3.7 L water after the first sampling), it is possible that conspecifics induced intensive bufadienolide production regardless of the presence or absence of predators, so that a further increase in toxin content in response to predators was either not necessary or physiologically not possible. However, in another mesocosm experiment with a similar tadpole density (1 tadpole/2.7 L water) we did find significant responses in bufadienolide synthesis to another stressor (Bókony et al., 2017). Therefore, it seems unlikely that tadpoles were physiologically unable to increase bufadienolide production in response to predator cues in the present experiment. Nonetheless, it is possible that tadpoles perceived toxin levels induced by density to provide enough protection from predators so a further increase was not necessary. Further experiments are needed to explicitly test this idea.

The predation trials revealed that tadpoles raised with dragonfly larvae survived better, compared to predator‐naïve tadpoles, when they were exposed to this predator. Because we could not detect any significant phenotypic responses induced by the presence of caged dragonflies during tadpole development (see also Appendix S1), we speculate that this treatment affected some unstudied aspect of behavior, morphology, physiology, or chemical defense of tadpoles (e.g., enhanced schooling behavior or elevated synthesis of nonbufadienolide defensive chemicals) that provided an effective defense against this predator. We did not observe differences in survival in predation trials between control tadpoles and their siblings raised with backswimmers, newts, or sticklebacks, similarly to earlier findings with various predators (McCollum & Van Buskirk, 1996; Van Buskirk & Relyea, 1998). However, when confronted with these three predators, especially the two vertebrate species, survival of toad tadpoles was very high (Figure 4), leaving little variation for a survival‐increasing effect of the rearing environment. Also, in case of newts and sticklebacks, toad tadpoles survived significantly better than common frog larvae, as demonstrated by the results of our feeding sessions in the rearing stage (Table 1) as well as the predation trials (Figure S4), irrespective of whether or not the toad tadpoles had been raised with predators. Although these predators might have avoided toad tadpoles for reasons other than toxicity, we observed sticklebacks to expel toad tadpoles after engulfing them (Henrikson, 1990; Kruse & Stone, 1984; Lawler & Hero, 1997; Peterson & Blaustein, 1991; Relyea, 2001a), indicating an aversion based on taste or chemical cues. Altogether, these findings suggest that the “baseline” toxin levels in the studied toad population (i.e., those expressed even in the absence of predators) are high enough to provide effective defense against newts and fishes. As mentioned above, this high baseline and the lack of plasticity may be due to permanent fish presence and/or high tadpole density in the natural habitat of this population.

We found that dragonflies consumed the most toad tadpoles, followed by backswimmers, sticklebacks, and newts in this order (Table 1, Figure 3). There was a marked difference between the effectiveness of invertebrates versus vertebrates, since during feeding sessions in the rearing stage of the current experiment, newts and sticklebacks consumed fewer of the offered naïve toad tadpoles than did backswimmers and dragonflies (Table 1). This differential susceptibility of toad tadpoles to invertebrate and vertebrate predators is consistent with earlier results showing that, typically, invertebrates find chemically defended tadpoles more palatable than do vertebrates (Gunzburger & Travis, 2005). This difference may, at least partly, be due to disparate sensitivity to the toxic effects of bufadienolides. Indeed, some species find bufadienolide‐containing prey unpalatable (Denton & Beebee, 1991; Henrikson, 1990; Kruse & Stone, 1984; Lawler & Hero, 1997; Peterson & Blaustein, 1991; Relyea, 2001a; Toledo & Jared, 1995), while others appear to be resistant to these compounds (Arbuckle, Rodríguez de la Vega, & Casewell, 2017; Dobler, Dalla, Wagschal, & Agrawal, 2012; Mohammadi et al., 2016; Ujvari et al., 2015), some of which are not known to be specialized predators of bufadienolide‐containing prey (Mohammadi et al., 2016). The high palatability of toad tadpoles to dragonfly larvae might be due to such a resistance. Furthermore, utilizing a special feeding apparatus may also circumvent chemical defenses of toad tadpoles: the pierce and suck feeding method of backswimmers may allow them to avoid the ingestion of bufadienolides produced and stored mainly in the skin of toads (Halliday et al., 2009; Toledo & Jared, 1995). On the other hand, species that engulf their entire prey and do not seem to have evolved resistance against bufadienolides, such as smooth newts and sticklebacks, likely become fully exposed to the toxic effects of tadpoles' chemical defenses upon ingestion.

We are highly confident that the lack of significant treatment effects in our experiment is not due to methodological shortcomings. A large number of studies using very similar methodology produced reliable results on inducible defenses in larval anuran amphibians (e.g. Van Buskirk, 2009; Hettyey et al., 2011, for a review see Wells, 2007). Also, previous studies exposing toad tadpoles specifically to very similar conditions, reported plastic phenotypic responses even under highly diluted concentrations of chemical cues from predators (in our experiment: one dragonfly/0.48 m^2^ in 130 L water vs. two crayfish and/or 1 trout/2.6 m^2^in 1,000 L water, Nyström & Åbjörnsson, 2000; or 2.2 dragonfly larvae/m^2^ in 560 L water, Van Buskirk, 2002a). Furthermore, we did observe a few treatment effects that are consistent with theoretical expectations. First, we found that the largest tadpoles raised in the presence of fish cues produced a lower number of bufadienolides at metamorphosis than expected. It is possible that such tadpoles maximized growth at the expense of bufadienolide synthesis to reach a size refuge against sticklebacks (Eklöv & Werner, 2000; Richards & Bull, 1990; Semlitsch & Gibbons, 1988). Second, tadpoles raised in the presence of dragonfly larvae enjoyed an enhanced survival probability as compared to their predator‐naïve sibs. Finally, we found that in the presence of sticklebacks, toad tadpoles metamorphosed earlier compared to control animals (Figure S3), which suggests that tadpoles perceived fish cues and reacted by enhancing allocation into development presumably to leave the dangerous waters as soon as possible (Chivers, Kiesecker, Marco, Wildy, & Blaustein, 1999; Laurila et al., 1998). These treatment effects together suggest that tadpoles did perceive the presence of predators during their development and were able to respond to them phenotypically; therefore, the lack of responses in chemical defenses was not due to an inability of tadpoles to sense olfactory cues on predation risk. The lack of increasing toxin content is also unlikely to be an artifact of an insensitivity of our chemical analytical framework, since the same build has proven to be effective in providing evidence for inducible bufadienolide synthesis in the same study species in the past (Bókony et al., 2017, 2018; Üveges et al., 2017).

Taken together, we did not find signs of inducible antipredator responses in the chemical defenses of common toad tadpoles. The observed level of chemical defense apparently provides protection from several vertebrate predators, while it defends less efficiently against invertebrates, which possibly are able to cope better with toad toxins. These results suggest that toad tadpoles currently may have the upper hand in the evolutionary arms race against some, but not all aquatic predators. Generally, the current study, with the addition of previous results, emphasizes that vertebrate chemical defenses may be influenced by a complex array of factors, including the evolutionary past of predator–prey coexistence, the predators' susceptibility to toxins, and prey's exposure to nonpredatory environmental stressors (Bókony et al., 2016, 2017, 2018; Üveges et al., 2017); therefore, the detection of inducible chemical defenses requires comprehensive understanding of this complexity.

## CONFLICT OF INTEREST

The authors declare no conflict of interest.

## AUTHOR CONTRIBUTIONS

AH and BÜ designed the study; BÜ, MS, and KM conducted the experiments; BÜ, MS, KM, VB, and AH took samples and measurements; BÜ prepared samples for chemical analysis; ÁMM and DK analyzed samples; BÜ and VB conducted statistical analyses; BÜ, VB, and AH wrote the manuscript with significant contributions from KM and HH. All authors read and approved the final manuscript.

## Data Availability

Datasets for the analysis of toxin content of toad tadpoles and survival in the predation trials (Appendix S2) are available in the figshare repository (https://figshare.com/s/4a35e739e62fc3cc814d, https://doi.org/10.6084/m9.figshare.5777550).
