# Supplementary material for: Chemical defense of toad tadpoles under risk by four predator species
Source: Ecol Evol. 2019 Apr 26;9(11):6287–99. doi: 10.1002/ece3.5202 (PMC6580299; doi:10.1002/ece3.5202)

Appendix 1

Chemical defense of toad tadpoles under risk by four predator species

Bálint Üveges, Márk Szederkényi, Katharina Mahr, Ágnes M. Móricz, Dániel Krüzselyi,

Veronika Bókony, Herbert Hoi, Attila Hettyey

Ecology and Evolution

Contents

Additional methods and results **2**

Diversity of bufadienolide compounds 2

Behavior 2

Morphology 3

Body mass 4

Length of larval development 5

Survival 5

Citations **6**

Table **7**

Table S1 7

Figures **8**

Fig. S1 8

Fig. S2 9

Fig. S3 10

Fig. S4 11

Additional methods and results

Here follows a brief summary of additional methods and results. Further details are available upon request from the authors.

Diversity of bufadienolide compounds

We quantified the diversity of bufadienolide compounds by applying hierarchical diversity partitioning using the 'hierDiversity' package in R (Marion *et al.*, 2015). Hierarchical diversity partitioning uses the concept of diversity indices, routinely applied in community ecology, to describe complex phenotypes (here, the whole bufadienolide mixture of toad tadpoles) as the diversity (effective number) of underlying phenotypical components (i.e. the individual bufadienolide compounds). Diversity is partitioned into an intra-individual (α) and an inter-individual (β) component. In our case, α is interpreted as the diversity of individual bufadienolide compounds of tadpoles, whereas β represents the diversity of distinct toxin profiles (i.e. bufadienolide "cocktails" found within individual tadpoles) among individuals. Using this approach, we found that the predator treatments did not affect the diversity of bufadienolide compounds in our study (Fig. S2).

Behavior

We observed behavior of toad tadpoles on days eleven, twelve and thirteen of the experiment. At the beginning of each day the mosquito netting was removed from the mesocosms, and two observers each counted the number of visible tadpoles and the number of active visible tadpoles in the mesocosms four or five times a day for the first two days, and three times on the third day. In total we obtained 25 observations of behavior for each mesocosm.

At each observation we registered the number of tadpoles that were visible, active and in the third of the mesocosm where the predator cage was located. For the analysis we calculated

daily mean percentage values of these numbers (N = 180 mesocosm means): we used the percentage of total number of tadpoles that were visible (visibility), the percentage of visible tadpoles that were active (activity) and the percentage of visible tadpoles in the third of the mesocosm where the predator cage was located (position). To calculate visibility we used the initial total number of tadpoles in each tub because overall survival was high in our experiment and treatment had no significant effect on survival (see section *Survival*). We analyzed the obtained percentages using linear mixed-effects models (LMM). Initial models included visibility, activity or position separately as the dependent variable, predator treatment as a fixed factor and family as random factor. We also included another random term in the models, with date as the random slope and mesocosm as the random intercept. We ran analyses in R 3.4.0 (R Development Core Team, 2017) using the 'lmer' function in the 'lme4' package (Bates *et al.*, 2015). *P*-values were calculated with the 'anova' function in 'lmerTest' (Kuznetsova *et al.*, 2017), using the Satterthwaite approximation for degrees of freedom. These analyses showed that the visibility ($F_{4,44}$ =1.046, p = 0.395), activity ($F_{4,45.44}$ =1.376, p = 0.257), and position ($F_{4,55}$ =0.954, p = 0.440) of tadpoles was not significantly affected by predator treatment.

Morphology

On day thirteen or fourteen of the experiment we haphazardly removed 8 toad tadpoles (developmental stage 29) from each mesocosm. After anesthetizing them with 0.05 w/w% MS-222 (tricaine methanesulfonate, Sigma-Aldrich), we placed them into a Plexiglas chamber filled with aged tap water. The chamber was part of a device that allowed us to take simultaneous photos from both the side and ventral views. From the photos we obtained the following body shape variables using ImageJ 1.46r (Schneider *et al.*, 2012): body length, body depth, body width, tail length, tail depth, tail muscle depth and tail muscle width.

We regressed each of the above body-shape variables against the square-root of body mass (transformation was applied to normalize the distribution of data), took the residuals from these regressions and used the mesocosm mean of these residuals for further analysis ($N = 60$ mesocosms). We analyzed morphology using a multivariate general linear model in IBM SPSS Statistics 20.0 (SPSS Inc., Chicago, USA), including the mesocosm mean of body shape residuals as dependent variables, and predator treatment and family as fixed factors. We found that predator treatment had no significant effect on the morphology of toad tadpoles (Wilk's λ = 0.743, $F_{24, 137.265}$= 0.509, $p = 0.972$).

Body mass

After we photographed the tadpoles we measured their body mass using an analytical balance to the nearest mg ($N = 480$ tadpoles). These animals were released at their pond of origin after the measurements were taken. Subsequently, we also measured the body mass of all animals remaining in the mesocosms on the day they started metamorphosis (developmental stage 42, $N = 1978$ metamorphs).

We analyzed body mass as the dependent variable in LMMs, using the 'lme' function in the R package 'nlme' (Pinheiro *et al.*, 2017). *P*-values were calculated using the 'anova' function in 'nlme'. In the model with tadpoles we entered predator treatment as fixed factor and mesocosm nested in family as random factors. In the model with metamorphs we also entered length of larval development (number of days from the start of the experiment to reaching developmental stage 42) and the number of surviving conspecifics in the same mesocosm as covariates. These analyses showed that predator treatment had no significant effect on the body mass of tadpoles (treatment: $F_{4,44}$ =1.736, $p = 0.159$) or metamorphs (full model: treatment: $F_{4,43}$ =1.125, $p = 0.304$; length of larval development: $F_{1,1917}$ =277.118, $p < 0.001$; number of

conspecifics: $F_{1,43}$ =1.814, p = 0.185; no variable became significant during stepwise model simplification).

Length of larval development

We analyzed development time (the median value of days needed to reach developmental stage 42 among all individuals in a mesocosm, N = 60 mesocosms) as the dependent variable, predator treatment as fixed factor and family as random factor in a LMM, using the 'lme' function in R. P-values were calculated using the 'anova' function in 'nlme'. Pairwise comparisons among treatment groups (comparing each predator-treatment group to the control group) were tested by calculating linear contrasts corrected for false discovery rate (Benjamini & Hochberg, 1995) in the 'lsmeans' package (Lenth, 2016) in R.

This analysis showed that predator treatment had a significant effect on the development time of toads ($F_{1,44}$ =3.355, p = 0.018): tadpoles that developed in the presence of sticklebacks metamorphosed on average 1.13 ± 0.345 (mean ± SE) days earlier than control animals (t_{44} = 3.265, p = 0.009; Fig. S3). Backswimmers (t_{44} = 1.572, p = 0.246), dragonfly larvae (t_{44} = 0.605, p = 0.631) and newts (t_{44} = 0.484, p = 0.631) did not have a significant effect on development time (Fig. S3).

Survival

We analyzed the survival of toad tadpoles in the rearing containers using a generalized linear mixed-effects model with quasi-binomial error distribution, using the 'glmmPQL' function in the 'MASS' package (Venables & Ripley, 2002). We entered the proportion of individuals surviving to developmental stage 42 (N = 60 mesocosms) as the dependent variable, predator treatment as fixed factor, development time (the mesocosm median of days needed to reach developmental stage 42) as covariate, and the treatment × development time interaction; and

we added family as random factor. *P*-values were calculated with the "Anova" function in the "car" package (Fox & Weisberg, 2011), using type-2 sum of squares.

Survival of toad tadpoles was overall very high in our study (mean: 94.29 %, 95% confidence interval: 89.86 – 98.94 %), and neither predator treatment ($\chi^2_4 = 2.70$, $p = 0.610$), nor development time ($\chi^2_1 = 0.33$, $p = 0.567$) had a significant effect. The treatment × development time interaction was also non-significant ($\chi^2_4 = 4.57$, $p = 0.334$).

Citations

Bates, D., Mächler, M., Bolker, B.M. & Walker, S. 2015. Fitting linear mixed-effects models using lme4. *J. Stat. Softw.* **67:** 1-48.

Benjamini, Y. & Hochberg, Y. 1995. Controlling the false discovery rate: a practical and powerful approach to multiple testing. *J. R. Stat. Soc. B: Stat. Methods* **57:** 289-300.

Fox, J. & Weisberg, S. 2011. *An R Companion to Applied Regression*, 2 edn. Sage Publications, Thousand Oaks, CA, USA.

Kuznetsova, A., Brockhoff, P.B. & Christensen, R.H.B. 2017. lmerTest Package: Tests in linear mixed effects models. *J. Stat. Softw.* **82:** 1-26.

Lenth, R.V. 2016. Least-squares means: the R package lsmeans. *J. Stat. Softw.* **69:** 1-33.

Marion, Z.H., Fordyce, J.A. & Fitzpatrick, B.M. 2015. Extending the concept of diversity partitioning to characterize phenotypic complexity. *Am. Nat.* **186:** 348-361.

Pinheiro, J., Bates, D., DebRoy, S., Sarkar, D. & R Core Team 2017. nlme: Linear and nonlinear mixed effects models, The R Project for Statistical Computing. R package version 3.1-131.

R Development Core Team 2017. A language and environment for statistical computing, R Foundation for Statistical Computing. Vienna, Austria.

Schneider, C.A., Rasband, W.S. & Eliceiri, K.W. 2012. NIH Image to ImageJ: 25 years of image analysis. *Nat. Methods* **9:** 671-675.

Venables, W.N. & Ripley, B.D. 2002. *Modern applied statistics with S.*, Fourth Edition edn. Springer, New York.

Table S1: Effects of age, dry mass, predator treatment and their interactions on the number of bufadienolide compounds (NBC) of common toad tadpoles (N = 118). We present the parameter estimates (± SE) of the final LMM model; the 'intercept' refers to control (predator-naïve) tadpoles and the first age class (developmental stage 29). Significant parameters are highlighted in bold.

	Estimate	SE	df	*t*	*p*
**intercept**	**12.765**	**0.788**	**52**	**16.204**	**<0.0001**
**age (stage 42)**	**-5.023**	**0.228**	**52**	**-22.017**	**<0.0001**
**dry mass (mg)**	0.145	0.052	52	2.755	0.008
dragonfly	1.099	0.968	44	1.135	0.262
backswimmer	1.181	0.940	44	1.256	0.216
**stickleback**	**1.934**	**0.926**	**44**	**2.090**	**0.043**
newt	-0.057	1.081	44	-0.053	0.958
dry mass × dragonfly	-0.071	0.061	52	-1.164	0.250
dry mass × backswimmer	-0.093	0.059	52	-1.579	0.121
**dry mass × stickleback**	**-0.139**	**0.056**	**52**	**-2.469**	**0.017**
dry mass × newt	0.001	0.069	52	0.012	0.990

Figures

Fig. S1: Alpha and beta diversity of bufadienolide compounds in toad tadpoles (developmental stage 29) and metamorphs (stage 42). Zero-order (q=0), first-order (q=1) and second-order (q=2) diversity indices, respectively, correspond to the number of toxin compounds (α) or toxin cocktails (β), Shannon entropy, and Simpson's probability of identity. Abbreviations of the treatments are as follows: C: control, D: dragonfly, B: backswimmer, S: stickleback, N: newt. Means and 95% confidence intervals are presented.

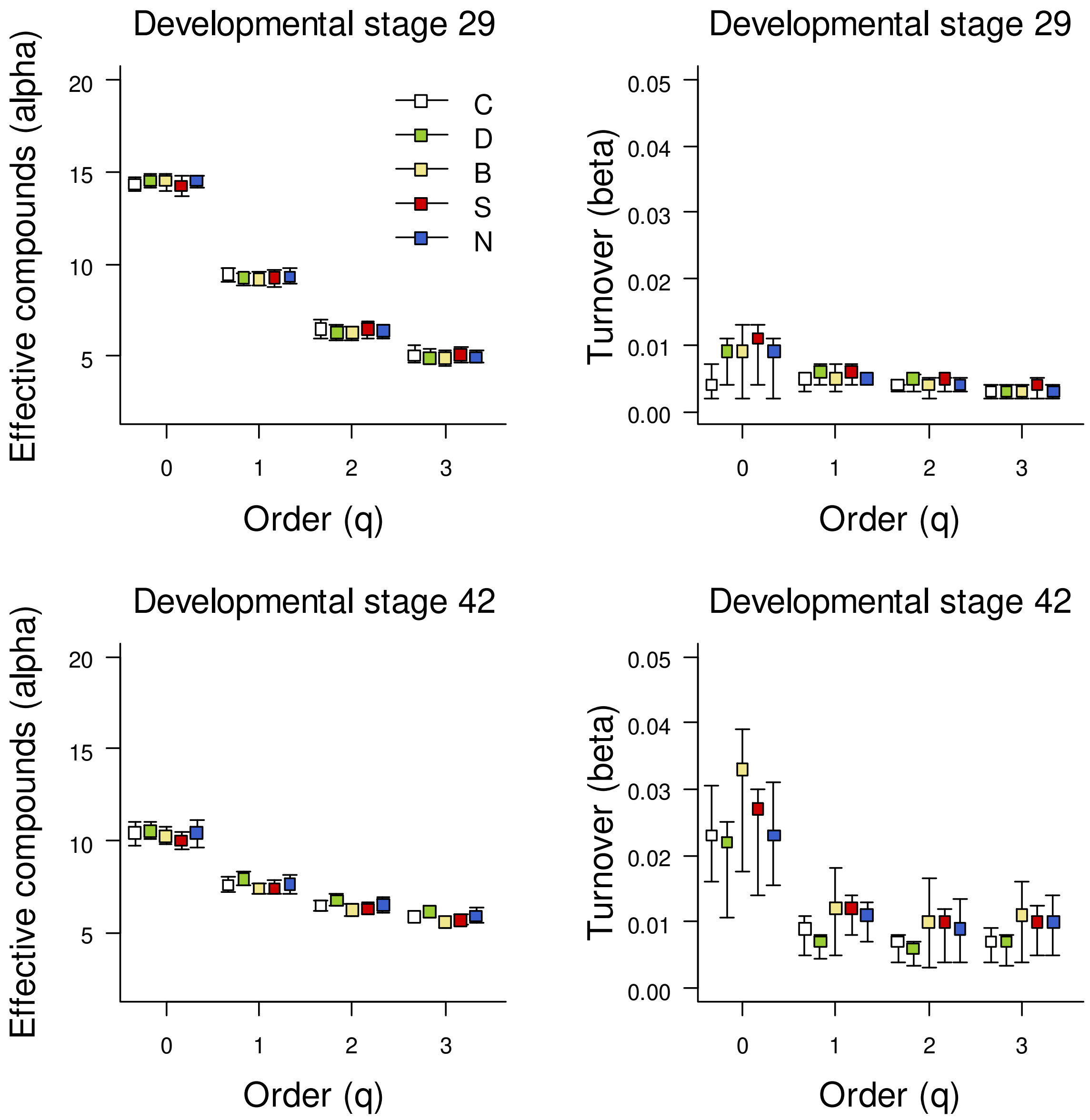

Fig. S2: Number of bufadienolide compounds (NBC) in relation to dry mass of toad tadpoles when reared without predators or in the presence of caged sticklebacks. Regression lines were fitted from the final model presented in Table S1 (the three-way interaction between age, dry mass and treatment was non-significant, see Table 2 in the main text).

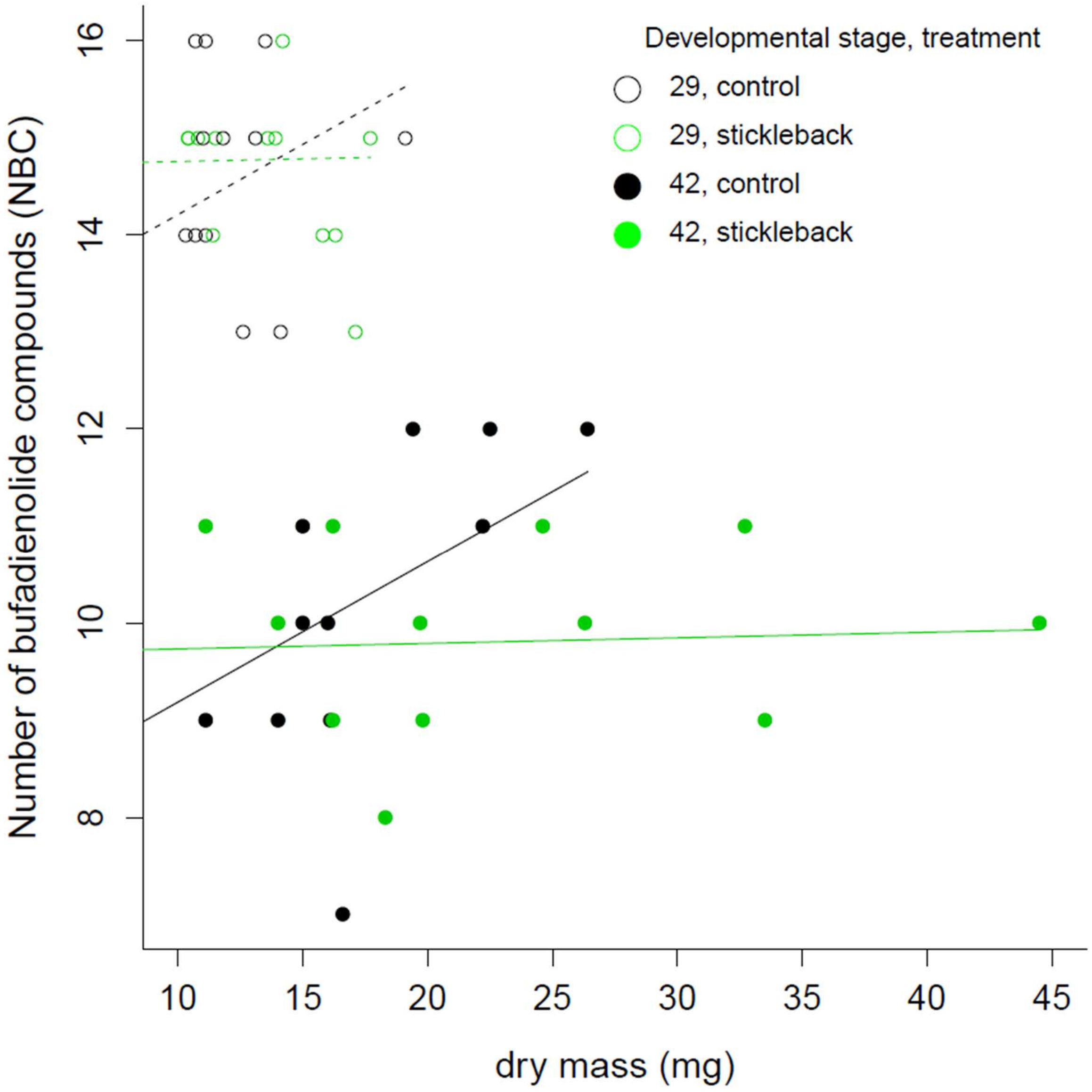

Fig. S3: Effect of predator treatment on length of larval development (mesocosm median number of days until metamorphosis) in common toad tadpoles. A significant difference between control tadpoles and tadpoles reared with fish is marked with asterisks ($p < 0.01$).

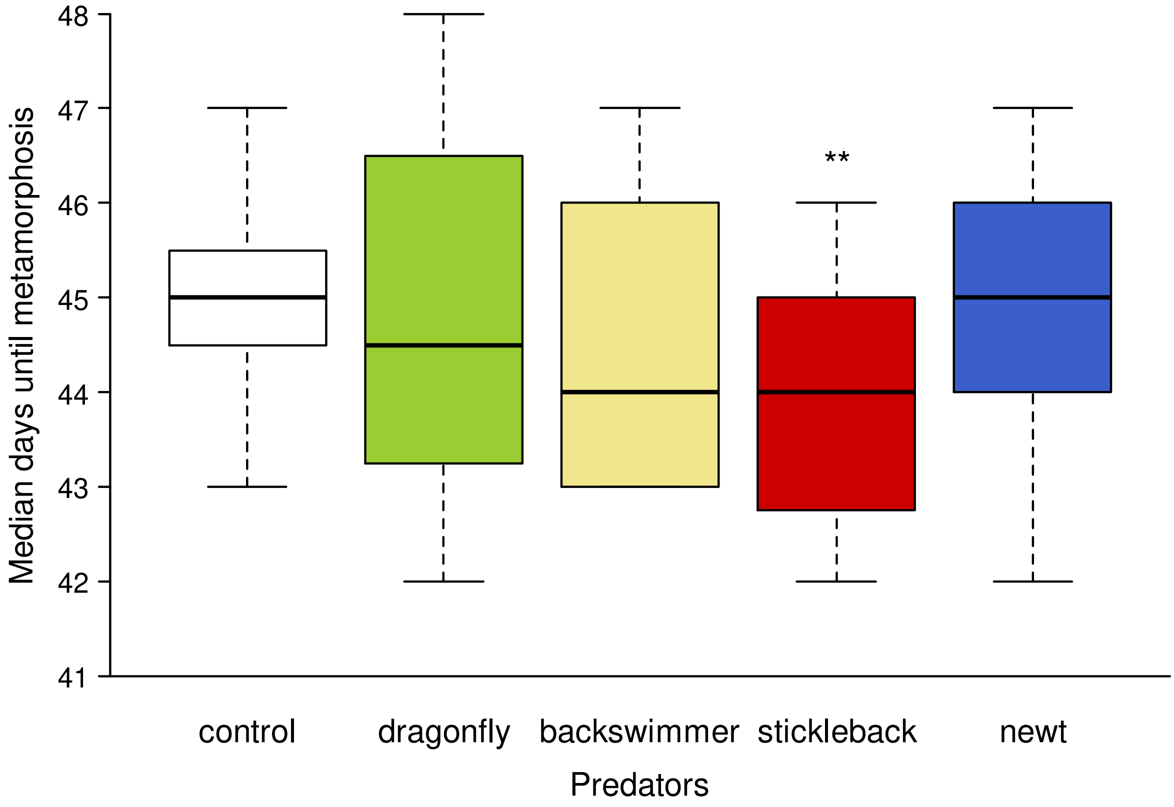

Fig. S4: Proportion of dead tadpoles (palatability) of common toads and common frogs in the predation trials. Significant differences between the two species are marked with asterisks (p = <0.001), a marginally non-significant difference is marked with a dagger symbol (p = 0.071).

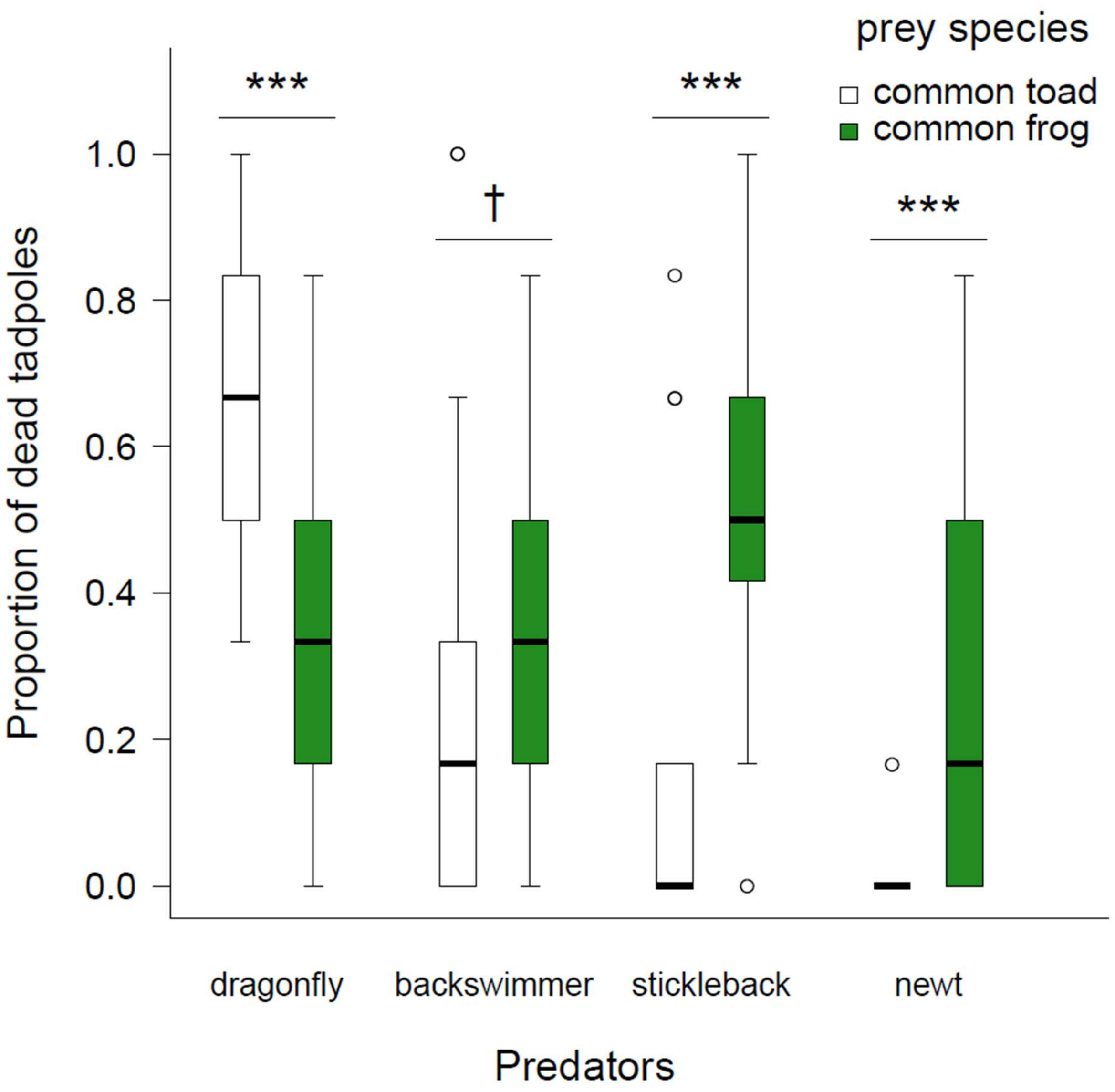

Supplement: Supplementary file 1 [file ECE3-9-6287-s001.pdf]
